# Spatial-dependent quantum dot-photon entanglement via tunneling effect

**DOI:** 10.1038/s41598-022-11810-8

**Published:** 2022-05-14

**Authors:** Yaser Delir Ghaleh Joughi, Mostafa Sahrai

**Affiliations:** Faculty of Physics, University of Tabriz, Tabriz, Iran

**Keywords:** Information theory and computation, Quantum physics

## Abstract

Utilizing the vortex beams, we investigate the entanglement between the triple-quantum dot molecule and its spontaneous emission field. We present the spatially dependent quantum dot-photon entanglement created by Laguerre-Gaussian (LG) fields. The degree of position-dependent entanglement (DEM) is controlled by the angular momentum of the LG light and the quantum tunneling effect created by the gate voltage. Various spatial-dependent entanglement distribution is reached just by the magnitude and the sign of the orbital angular momentum (OAM) of the optical vortex beam.

## Introduction

Intrinsic quantum correlation between different parts of a system leads to a quantum phenomenon called entanglement^1^. Entanglement is a foundation of quantum information processing and quantum computing^2^, quantum transport^3–5^, quantum dense coding^6^, and quantum cryptography^7,8^. Among many proposals in the entanglement between particles of a quantum system, the generation of atom-photon entanglement has reached a great deal of attention in the last two decades. It is well known that atom-photon entanglement has essential applications in quantum information theory, such as quantum repeaters and quantum networks^9^, that is widely investigated both experimentally and theoretically^10–12^. Coherent control of spontaneous emission leads to the creation of the controllable entanglement between the atom and its spontaneous emission field in ${\Lambda }$^13^, and *V*-type^14^ atomic systems. Maximally atom-photon entanglement has also been reached by the rate of an incoherent pump field and the quantum interference created by spontaneous emission and an incoherent pump field^15^. The effect of the surrounding environment such as photonic crystal on spontaneous emission and consequently dynamical behavior of the atom-photon entanglement is proposed^16^. In another proposal, the entanglement between the quantum dot molecule and its spontaneous emission field is also discussed at the rate of an incoherent pump field^17^. In all the above proposals, the entanglement between the atom (or quantum dot molecule) and its spontaneous emission field is coherently controlled by the laser fields or even by the incoherent pump field. It is interesting to generate a spatially dependent entanglement between the atom and its spontaneous emission. In this article, using an optical vortex beam, we produce spatially dependent quantum dot-photon entanglement. An optical vortex beam is a nonzero orbital angular momentum (OAM) beam with a helical wavefront and phase term as that is a function of azimuthal coordinate^18,19^. The interaction of an atom with a vortex beam has widely been proposed. Light-induced torque on moving atoms by the vortex beam^20^, slow light by the vortex beam^21^, and transfer and storage of the vortex state in light and matter waves^22^ are also discussed. OAM-based four-wave mixing^23^, spatially dependent optical transparency^24,25^, and entanglement of OAM states of photon pairs^26^ are the other potential applications of the vortex beam. Vortex slow light^27^ provides another issue for manipulating the optical information during slow light storage and retrieval^28^. The freedom action of the phase rotation in the vortex beam, which manifests itself in orbital angular momentum, leads to an increase in the system capacity and the spectral efficiency of wireless communication in millimeter waves^29^. A vortex beam was utilized to obtain images with a spatial resolution higher than the natural diffraction limit, i.e., stimulated emission depletion microscopy^30^. LG beam is a form of vortex beam with a helical phase structure and a doughnut-like intensity profile. This beam has recently been employed to narrow the Doppler line-shapes^31^, atom localization^32^, and spatially dependent atom-photon entanglement^33^. Such a beam was also exercised for generating entanglement of the orbital angular momentum states of photons^34^, quantum cryptography^35^, and a free-space quantum key distribution mechanism^36^. Further, orbital angular momentum can be considered as a suitable degree of freedom to increase the communication capacity of open space^37^, and the transmission and processing of quantum information^38^.

In this paper, we propose the entanglement between the triple quantum dot molecule and its spontaneous emission field using optical vortex beams. We present the spatially dependent quantum dot-photon entanglement created by LG fields. The effect of controlling parameters such as field intensity and tunneling effect on the entanglement of quantum dot molecule and its spontaneous emission field is then discussed. We prove that the DEM completely depends on the position of space points, magnitude, and the sign of orbital angular momentum (OAM) of the optical vortex beam.

In Sect. 2, we present the quantum dot molecule and the related equations to propose the degree of entanglement. We discuss the theoretical results and their physical mechanism in Sect. 3. The paper concludes in Sect. 4.

## Model and equations

**Figure 1:**
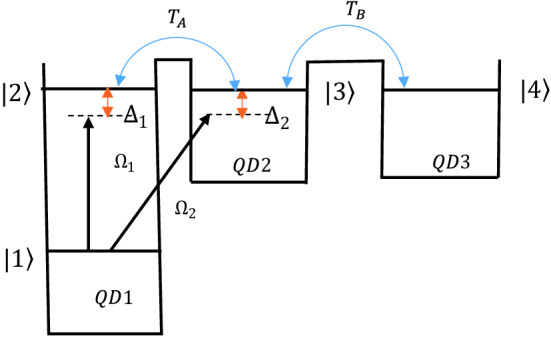
A four-level quantum dot molecule's band diagram. A triple quantum dot molecule is made up of three dots (“QD 1,” “QD 2,” and “QD 3”) with different tunneling rates $T_{A}$ and $T_{B}$. The corresponding energy level structure is denoted by levels $\left| 1 \right\rangle$, $\left| 2 \right\rangle , \left| 3 \right\rangle ,$ and $\left| 4 \right\rangle$.

Consider a quantum dot molecule with three dots and various band structures, i.e. QD1, QD2, and QD3 as shown in Fig. 1. The three dots are coupled to each other via electron tunneling, where the intra-dot tunneling between “QD 1- QD 2” and “QD 2- QD 3” are denoted by tunneling rates $T_{A}$ and $T_{B}$, respectively^17^.

This quantum dot molecule is similar to a four-level atomic system as depicted in Fig. 1. The lower and higher conducting band levels of the left quantum dot are displayed by $$\left| 1 \right\rangle$$ and $$\left| 2 \right\rangle$$, whereas the excited conducting band levels of the second and third quantum dots are denoted by $$\left| 3 \right\rangle$$ and $$\left| 4 \right\rangle$$, respectively. The energy difference between the three upper levels and the lowest level is so large, thus their tunneling couplings are neglected. Levels $$\left| 3 \right\rangle$$ and $$\left| 4 \right\rangle$$ become close to the level $$\left| 2 \right\rangle$$ if a gate voltage is applied, while level $$\left| 1 \right\rangle$$ still has a large energy difference with the upper levels. A gate electrode placed between nearby quantum dots can adjust the tunnel barrier in triple quantum dots. When two laser fields resonantly couple the lower level $$\left| 1 \right\rangle$$ to upper levels $$\left| 2 \right\rangle$$ and $$\left| 3 \right\rangle$$ in QD1 and QD2, an electron excites from level $$\left| 1 \right\rangle$$ to the superposition of the upper levels $$\left| 2 \right\rangle$$ and $$\left| 3 \right\rangle$$. For electron transition from level $$\left| 1 \right\rangle$$ to level $$\left| 3 \right\rangle$$, the thin barrier thickness of QD1 and QD2 is required. In addition, tunneling allows an electron also to be transformed from QD1 to QD2 and from QD2 to QD3 by tunneling rates $$T_{A}$$ and $$T_{B}$$. As an experimental example, a GaAs/AlGaAs heterostructure with a two-dimensional electron gas situated 470 Å below the surface and a sheet density of 3.7 × 10^11^ cm^−2^ and mobility 5 × 10^5^ cm^2^/Vs at 10 K, and phase coherence length > 20 μm for $$T < 1 K $$ can be used to create triple quantum dot samples^39^.

In the following discussion, we consider an ensemble of such four-level quantum dot molecules, and investigate the spatial-dependent degree of entanglement (DEM) as depicted in Ref.^33^ for atomic vapors. Thus, position dependent entanglement is presented via using various modes of the LG beam. The proposed coordinates x and y define as a point on the plane x–y perpendicular to the propagation direction of the LG beam.

Now, we present the Hamiltonian for the quantum-dot molecule described in Fig. 1. The Hamiltonian in Shrodinger picture is described as

1 $$ {\mathcal{H}} = {\mathcal{H}}_{0} + {\mathcal{H}}_{1} + {\mathcal{H}}_{2} . $$

     Here, $H_{0}$ denotes the free energy part, while $H_{1}$ is the interaction Hamiltonian of the QD system with the LG fields. The term $H_{2}$ denotes the electron tunneling processes. Then tunneling effect may transfer the electron from QD1 to QD2 and QD3. Perturbation theory can be used to describe electron tunneling in a barrier, as demonstrated by Bardeen's technique^40^. The tunneling rate between two quantum dots can be controlled by the applied current, voltage, and thickness of the potential barrier. We assume that the coupling LG field of frequency $\nu_{1}$ is applied in the transition $\left| 1 \right\rangle \leftrightarrow \left| 2 \right\rangle$, while another LG field of frequency $\nu_{2}$ drives transition $\left| 1 \right\rangle \leftrightarrow \left| 3 \right\rangle$. To examine the position-dependent quantum-dot photon entanglement, we define the LG field with angular frequency $\omega_{LG}$, and Rabi-frequency ${\Omega }_{1} = \vec{\mu }_{21} .u_{1} /\hbar$ and ${\Omega }_{2} = \vec{\mu }_{31} .u_{2} /\hbar$. Here, $u_{j}^{ } \left( {j = 1, 2} \right)$ denote the amplitude of the $LG$ beams that define as

2 $$ \begin{aligned} u_{j} \left( {r.\phi .z} \right) = & \frac{{c_{p}^{{l_{j} }} w_{0} }}{w\left( z \right)}\left( {\frac{\sqrt 2 r}{{w\left( z \right)}}} \right)^{{\left| {l_{j} } \right|}} e^{{\frac{{ - r^{2} }}{{w^{2} \left( z \right)}}}} L_{p}^{{\left| {l_{j} } \right|}} \left[ {\frac{{2r^{2} }}{{w^{2} \left( z \right)}}} \right] \\ & \quad \times \exp \left[ { - \frac{{ikr^{2} }}{2R\left( z \right)} + i\left( {2p + \left| {l_{j} } \right| + 1} \right)arctan\left( {\frac{z}{{z_{R} }}} \right)} \right] \times {\text{exp}}\left[ { - il_{j} \phi } \right],\;\left( {j = 1, 2} \right) \\ \end{aligned} $$

where $$r,\phi ,z$$ are cylindrical coordinates. The parameters *l* and *p* are LG beam mode parameters, which represent the azimuthal mode index and the radial index, respectively. The value of $$ p$$ is set to be identical constant value. Here,$$ z_{R} = \frac{{kw_{0}^{2} }}{2}$$ and $$tan^{ - 1} \left( \xi \right)$$ are the Riley length and the phase displacement. In addition,$$ w\left( z \right) = w_{0} \sqrt {1 + { }\xi^{2} }$$ and $$r = \sqrt {x^{2} + { }y^{2} }$$ are beam waist in distance $$z$$, and radial cylindrical, respectively. Here $$ w_{0}$$ is beam waist in $$z = 0.$$ The parameter $$L_{p}^{{\left| {l_{j} } \right|}} \left( {\frac{{2r^{2} }}{{w^{2} \left( z \right)}}} \right)$$ represents the generalized Laguerre polynomial, $$C_{p}^{l}$$ is a required normalization constant, and $$arctan^{ } \left( {\frac{z}{{z_{R} }}} \right)$$ shows the Gouy phase at position z. The radius curvature of the wavefront R(z) in the spherical approximation is expressed as

3 $$ {\text{R}}\left( {\text{z}} \right) = {\text{z}}\left( {1 + \left( {\frac{{{\text{z}}_{{\text{R}}} }}{{\text{z}}}} \right)^{2} } \right).{ } $$

At point $z = 0$, the amplitude of the LG beam converts to

4 $$ u_{j}^{ } \left( {r.\phi .0} \right) = c_{p}^{l} \left( {\frac{\sqrt 2 r}{{w_{0} }}} \right)^{{\left| {l_{j} } \right|}} e^{{\frac{{ - r^{2} }}{{w_{0}^{2} }}}} L_{p}^{{\left| {l_{j} } \right|}} \left[ {\frac{{2r^{2} }}{{w_{0}^{2} }}} \right]\exp \left[ { - il_{j} \phi } \right], $$

and the transverse view of LG beam intensity is

5 $$ {\text{I}}\left( {\text{r}} \right) = {\text{I}}_{0} \exp \left( { - \frac{{2{\text{r}}^{2} }}{{{\text{w}}_{0} }}} \right)\left( {\frac{{2{\text{r}}^{2} }}{{{\text{w}}_{0}^{2} }}} \right)^{{\left| {{\text{l}}_{{\text{j}}} } \right|}} \left( {{\text{L}}_{{\text{p}}}^{{\text{l}}} \left( {\frac{{2{\text{r}}^{2} }}{{{\text{w}}_{0}^{2} }}} \right)} \right)^{2} . $$

Here ${\text{I}}_{0}$ is the normalized intensity of the LG beam. Now the detailed form of Eq. (1) is given by

6 $$   \begin{gathered}   {\mathcal{H}}_{0}  = \hbar \omega _{1} \left| 1 \right\rangle \left\langle 1 \right| + \hbar \omega _{2} \left| 2 \right\rangle \left\langle 2 \right| + \hbar \omega _{3} \left| 3 \right\rangle \left\langle 3 \right| + \hbar \omega _{4} \left| 4 \right\rangle \left\langle 4 \right|, \hfill \\   {\mathcal{H}}_{1}  =  - \frac{1}{2}\hbar \Omega _{1} e^{{ - i\nu _{1} t}} \left| 2 \right\rangle \left\langle 1 \right| - \frac{1}{2}\hbar \Omega _{1}^{*} e^{{ - i\nu _{1} t}} \left| 1 \right\rangle \left\langle 2 \right| - \frac{1}{2}\hbar \Omega _{2} e^{{ - i\nu _{2} t}} \left| 3 \right\rangle \left\langle 1 \right| - \frac{1}{2}\hbar \Omega _{2}^{*} e^{{ - i\nu _{2} t}} \left| 1 \right\rangle \left\langle 3 \right| \hfill \\    {\mathcal{H}}_{2}  = \frac{1}{2}\hbar T_{A} \left| 2 \right\rangle \left\langle 3 \right| + \frac{1}{2}\hbar T_{A} \left| 3 \right\rangle \left\langle 2 \right| + \frac{1}{2}\hbar T_{B} \left| 3 \right\rangle \left\langle 4 \right| + \frac{1}{2}\hbar T_{B} \left| 4 \right\rangle \left\langle 3 \right| \prime. \hfill \\ \end{gathered}   $$

Here $\hbar \omega_{i}$ is the energy of level $\left| i \right\rangle$, and ${\Omega }_{1} \left( {{\Omega }_{2} } \right)_{ }$ represent the Rabi-frequency between levels $|1 \leftrightarrow |2$ $\left( {|1 \leftrightarrow |3} \right)$.

The density matrix elements in an arbitrary multilevel QDs system can be obtained using the Liouville equation as

7 $$ \frac{\partial \rho }{{\partial {\text{t}}}} = - \frac{i}{\hbar }\left[ {H,\rho } \right] + L_{\rho } , $$

where $L_{\rho }$ represents the relaxation mechanisms such as spontaneous emission and pure dephasing terms that are added to the density matrix through $L_{\rho } = \mathop \sum \limits_{j = 2}^{4} ({\upgamma }_{j1} D\left[ {\sigma_{1j} } \right]\rho + \frac{{\gamma_{j}^{dph} }}{2}D\left[ {\sigma_{jj} } \right]\rho ),$ where $D\left[ {\sigma_{ij} } \right]\rho = \left( {1/2} \right)\left( {2\sigma_{ij} \rho \sigma_{ji} - \sigma_{ji} \sigma_{ij} \rho - \rho \sigma_{ji} \sigma_{ij} } \right)$^44^. Here, ${\upgamma }_{j1}$ and $\gamma_{j}^{dph}$ represent the radiative decay and pure dephasing rates, respectively. In addition $\sigma_{ij} = |ij|$ denote the transition operates. Thus, all the incoherent processes that appeared in the density matrix element are imported through the parameters $L_{\rho }$ defined in Eq. (7).

Substituting Eqs. (6) in Eq. (7) provides the density matrix elements as

8 $$ \begin{aligned} & \dot{\tilde{\rho }}_{11} = - \frac{1}{2}i{\Omega }_{1} \tilde{\rho }_{12} - \frac{1}{2}i{\Omega }_{2} \tilde{\rho }_{13} + \frac{1}{2}i{\Omega }_{1}^{*} \tilde{\rho }_{21} + {\upgamma }_{21} \tilde{\rho }_{22} + \frac{1}{2}i{\Omega }_{2}^{*} \tilde{\rho }_{31} + {\upgamma }_{31} \tilde{\rho }_{33} + {\upgamma }_{41} \tilde{\rho }_{44} , \\ & \dot{\tilde{\rho }}_{12} = - \frac{1}{2}i{\Omega }_{1}^{*} \tilde{\rho }_{11} - \left( {i{\Delta }_{1} + {\Gamma }_{21} } \right)\tilde{\rho }_{12} + \frac{1}{2}iT_{A} \tilde{\rho }_{13} e^{ - i\delta t} + \frac{1}{2}i{\Omega }_{1}^{*} \tilde{\rho }_{22} + \frac{1}{2}i{\Omega }_{2}^{*} \tilde{\rho }_{32} , \\ & \dot{\tilde{\rho }}_{13} = - \frac{1}{2}i{\Omega }_{2}^{*} \tilde{\rho }_{11} + \frac{1}{2}iT_{A} \tilde{\rho }_{12} e^{i\delta t} - \left( {i{\Delta }_{2} + {\Gamma }_{31} } \right)\tilde{\rho }_{13} + \frac{1}{2}iT_{B} \tilde{\rho }_{14} + \frac{1}{2}i{\Omega }_{1}^{*} \tilde{\rho }_{23} + \frac{1}{2}i{\Omega }_{2}^{*} \tilde{\rho }_{33} , \\ & \dot{\tilde{\rho }}_{14} = \frac{1}{2}iT_{B} \tilde{\rho }_{13} - \left( {i\left( {{\Delta }_{2} - \omega_{43} } \right) + {\Gamma }_{41} } \right)\tilde{\rho }_{14} + \frac{1}{2}i{\Omega }_{1}^{*} \tilde{\rho }_{24} + \frac{1}{2}i{\Omega }_{2}^{*} \tilde{\rho }_{34} , \\ & \dot{\tilde{\rho }}_{22} = \frac{1}{2}i{\Omega }_{1} \tilde{\rho }_{12} - \frac{1}{2}i{\Omega }_{1}^{*} \tilde{\rho }_{21} - {\upgamma }_{21} \tilde{\rho }_{22} + \frac{1}{2}iT_{A} \tilde{\rho }_{23} e^{ - i\delta t} - \frac{1}{2}iT_{A} \tilde{\rho }_{32} e^{i\delta t} , \\ & \dot{\tilde{\rho }}_{23} = \frac{1}{2}i{\Omega }_{1} \tilde{\rho }_{13} - \frac{1}{2}i{\Omega }_{2}^{*} \tilde{\rho }_{21} + \frac{1}{2}iT_{A} \tilde{\rho }_{22} e^{i\delta t} + \left( {i\left( {{\Delta }_{1} - {\Delta }_{2} } \right) - {\Gamma }_{32} } \right)\tilde{\rho }_{23} + \frac{1}{2}iT_{B} \tilde{\rho }_{24} - \frac{1}{2}iT_{A} \tilde{\rho }_{33} e^{i\delta t} , \\ & \dot{\tilde{\rho }}_{24} = \frac{1}{2}i{\Omega }_{1} \tilde{\rho }_{14} + \frac{1}{2}iT_{B} \tilde{\rho }_{23} + \left( {i\left( {{\Delta }_{1} - {\Delta }_{2} + \omega_{43} } \right) - {\Gamma }_{42} } \right)\tilde{\rho }_{24} - \frac{1}{2}iT_{A} \tilde{\rho }_{34} e^{i\delta t} , \\ & \dot{\tilde{\rho }}_{33} = \frac{1}{2}i{\Omega }_{2} \tilde{\rho }_{13} - \frac{1}{2}iT_{A} \tilde{\rho }_{23} e^{ - i\delta t} - \frac{1}{2}i{\Omega }_{2}^{*} \tilde{\rho }_{31} + \frac{1}{2}iT_{A} \tilde{\rho }_{32} e^{i\delta t} - {\upgamma }_{31} \tilde{\rho }_{33} + \frac{1}{2}iT_{B} \tilde{\rho }_{34} - \frac{1}{2}iT_{B} \tilde{\rho }_{43} , \\ & \dot{\tilde{\rho }}_{34} = \frac{1}{2}i{\Omega }_{2} \tilde{\rho }_{14} - \frac{1}{2}iT_{A} \tilde{\rho }_{24} e^{ - i\delta t} + \frac{1}{2}iT_{B} \tilde{\rho }_{33} + \left( {i\omega_{43} - {\Gamma }_{43} } \right)\tilde{\rho }_{34} - \frac{1}{2}iT_{B} \tilde{\rho }_{44} , \\ & \tilde{\rho }_{11} + \tilde{\rho }_{22} + \tilde{\rho }_{33} + \tilde{\rho }_{44} = 1, \\ \end{aligned} $$

where we applied the rotating frame transformation as $\rho_{12} = \tilde{\rho }_{12} e^{{i\nu_{1} t}}$, $\rho_{13} = \tilde{\rho }_{13} e^{{i\nu_{2} t}}$, $\rho_{14} = \tilde{\rho }_{14} e^{{i\nu_{2} t}}$, $\rho_{23} = \tilde{\rho }_{23} e^{{ - i\left( {\nu_{1} - \nu_{2} } \right)t}}$ and $,\rho_{24} = \tilde{\rho }_{24} e^{{ - i\left( {\nu_{1} - \nu_{2} } \right)t}}$. Here, $\rho_{ij} = \left| i \right\rangle \left\langle j \right|$ $\left( {i,j{ } = 0,{ }1,{ }2,{ }3{ }i \ne j} \right)$ are the coherence term, and $\rho_{ii} = \left| i \right\rangle \left\langle i \right|\left( {i = 1,{ }2,{ }3,{ }4} \right)$ represent the population. Detuning parameters defined by the ${\Delta }_{1} = \nu_{1} - \omega_{21}$ and ${\Delta }_{2} = \nu_{2} - \omega_{31} ,$ where the frequency difference between two applied fields is $\nu_{1} - \nu_{2} = \delta$. The terms $\omega_{ij} { }\left( {i,j = 1,{ }2,{ }3,{ }4{ }i \ne j} \right)$ declare as the frequency of the transitions $\left| i \right\rangle \leftrightarrow \left| j \right\rangle$. The total decay rates, ${\Gamma }_{ij} { }\left( {i \ne j} \right)$, are given by ${\Gamma }_{21} = \frac{{\gamma_{21} }}{2} + \gamma_{21}^{dph} ,{{ \Gamma }}_{31} = \frac{{\gamma_{31} }}{2} + \gamma_{31}^{dph}$, ${\Gamma }_{41} = \frac{{\gamma_{41} }}{2} + \gamma_{41}^{dph}$, ${\Gamma }_{32} = \frac{{\gamma_{{21 + \gamma_{31} }} }}{2} + \gamma_{32}^{dph} ,{{ \Gamma }}_{42} = \frac{{\gamma_{21} + \gamma_{41} }}{2} + \gamma_{42}^{dph} \;{\text{and}}\;{\Gamma }_{43} = \frac{{\gamma_{31} + \gamma_{41} }}{2} + \gamma_{43}^{dph} ,{ }$ which are determined by electron–electron interface roughness, phonon scattering processes, and $\gamma_{ij}^{dph}$ are the dephasing rate of quantum coherence in $\left| i \right\rangle \leftrightarrow \left| j \right\rangle$ pathway. The spontaneous emission from level $\left| i \right\rangle { }\left( {i = 2,3,4} \right)$ to level $\left| 1 \right\rangle$ is also denoted by $\gamma_{i1} .$

Now, we will determine the entanglement between the quantum dot molecule and its spontaneous emission field via the quantum entropy. Von-Neumann entropy utilizes for determining the DEM as

9 $$ S_{i} \left( t \right) = - Tr\left( {\rho_{i} Log_{2} \rho_{i} } \right),\;\left( {i = d,f} \right) $$

where $\rho$ is the reduced density matrix operator. Here $d$ and $f$ stand for quantum dot molecule and photon. The total entropy of the quantum-dot molecule and the spontaneous emission field is also determined by^41^

10 $$ S_{d\left( f \right)} = - Tr\left( {\rho_{d\left( f \right)} Log_{2} \rho_{d\left( f \right)} } \right). $$

To measure the degree of entanglement of a pure state $\rho ,$ we only need the quantum dot molecule entropy $S_{d} \left( t \right)$, given in Eq. (9). The quantum-dot molecule entropy can also be represented according to the term of eigenvalues $\lambda_{i} \left( t \right)$ of the reduced density operator as a DEM. The degree of quantum dot-photon entanglement is then given by

11 $$ DEM\left( t \right) = S_{d} \left( t \right) = S_{f} \left( t \right) = - \mathop \sum \limits_{i = 1}^{N} \lambda_{i} \log_{2} \lambda_{i} , $$

where $\lambda_{i}$ denotes the eigenvalues of the reduced density matrix operator.

Note that Von Neumann entropy can be interpreted as a measure of the unpredictability of a quantum state measurement. For calculating the DEM of subsystem, the reduced density matrix should be calculated, where the reduced density matrix is the entropy of studied subsystem. The entropy is defined as a measure of the system's disorder. The higher entropy of quantum system, the higher unpredictability or entanglement. So, the von-Neumann entropy of the reduced density matrix in the studied QDM is sufficient to quantify the degree of entanglement.

Note that in a realistic case the whole combined system is including QDM, photonic subsystem, i.e. free field modes due to spontaneous emission, and pure dephasing due to incoherent processes such as phonon scattering. So the whole state should be in a mixed state. This may destroy the coherence mechanisms in a realistic case even at low temperature, however slightly different choice of the pure dephasing model following Ref.^42,43^ does not qualitatively change our results.

Here, in order to obtain a quantum pure state, we consider the whole combined system of interest includes QDM and free fields created by spontaneous emission. Thus, we consider all the quantum dots initially in their ground states. In this case the total entropy of this pure system $S_{df}$ is time independent due to the unitary time evolution of the reduced density operators $\rho_{d} { }$ and ${ }\rho_{f}$. Then, by calculating the total entropy of the system, it is impossible to investigate the entanglement of quantum dots and its spontaneous emission. However, the reduced entropies, $S_{d}$ and $S_{f}$ , are time dependent and they can evaluate from a pure state to a mixed or maximally mixed state. Consequently, the total pure state becomes entangled and cannot be written as a tensor product of the two subsystems. Therefore, we utilize the reduced quantum entropy as a measure of the degree of entanglement between the quantum dots and its spontaneous emission field^15,44^.

## Results and discussion

**Figure 2:**
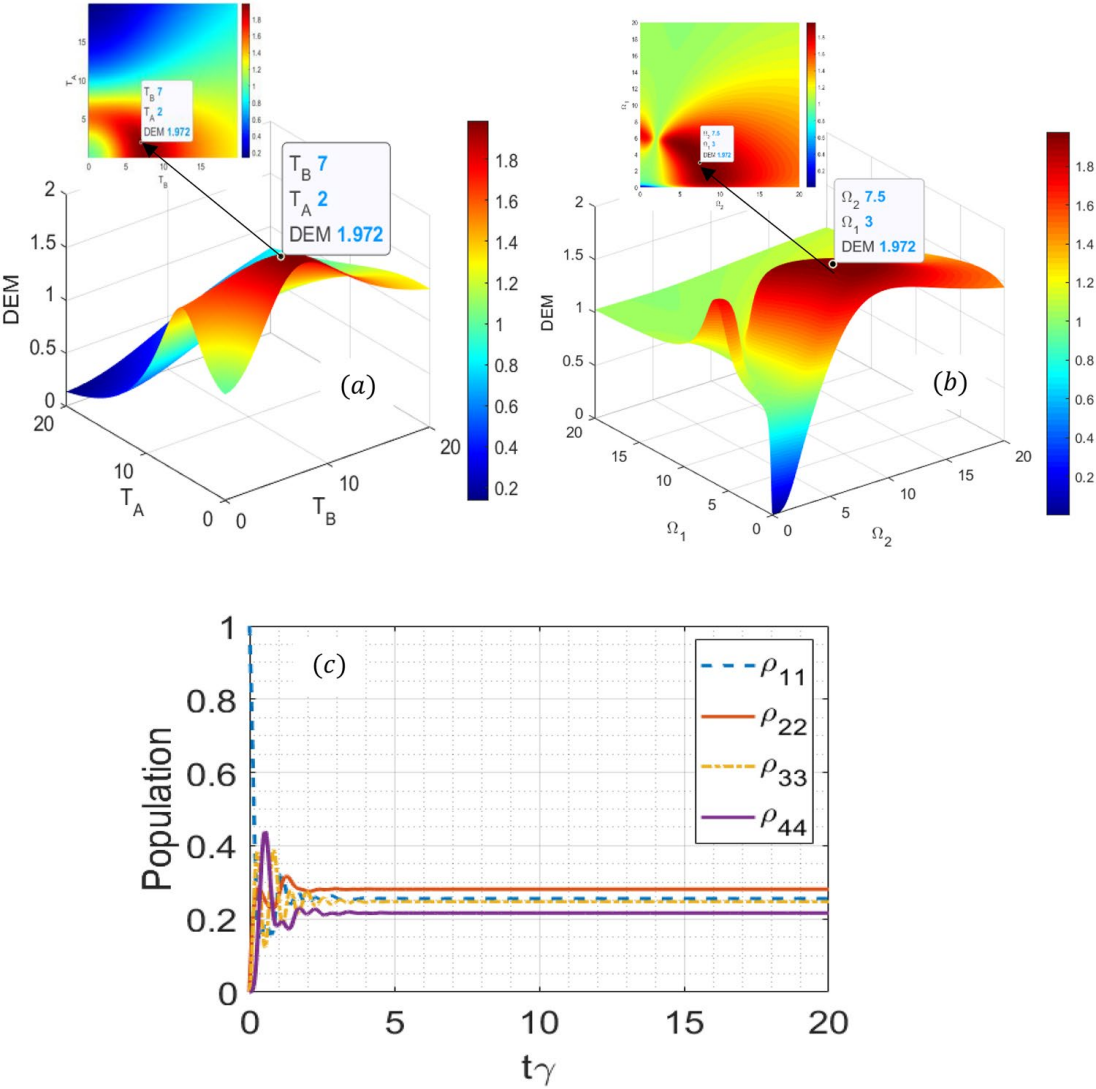
(**a**) DEM plots as a function of $T_{A}$ and $T_{B}$. Parameters are ${\Omega }_{1} = 3 \gamma$, and ${\Omega }_{2} = 7.5 \gamma .$ (**b**) DEM plots as a function of ${\Omega }_{1}$ and ${\Omega }_{2}$ for $T_{A} = 2{\upgamma }$, and $T_{B} = 7{\upgamma }.$ (**c**) Evolution of the population distribution a function of normalized time $t\gamma$ for $\Omega_{1} = 3{ }\gamma ,\Omega_{2} = 7.5 \gamma$, $T_{A} = 2{\upgamma }$, and $T_{B} = 7{\upgamma }.$ Other parameters are $\delta = {\Delta }_{1} = {\Delta }_{2} = 0$ and $\omega_{43} = 0.6 \gamma$*.*

Now, we analyze the position-dependent entanglement between the quantum dot molecule and its spontaneous emission using the LG beam. For a paraxial beam such as the Laguerre Gaussian wave, the angular momentum can be divided into two categories: spin angular momentum relates to polarization and orbital angular momentum which is due to the spatial distribution of the wavefront^45^. The entanglement of the quantum dot-photon system depends on the intensity profile and the orbital angular momentum of the applied LG field. So different angular momentum implies various entanglement designs depending on spatial locations. Density matrix Eqs. (8) along with Eqs. (2) and (11) are numerically solved to reach the optimal $DEM$. We chose the typical decay rate $\hbar \gamma_{ } = 10{{ \mu { \text{eV}} }}$, and all the parameters are scaled in a unit of $\gamma_{ }$^46^. Thus, the total decay and the spontaneous emission rates are ${\Gamma }_{21} = 1.6\gamma_{ } ,{{ \Gamma }}_{32} = 0.05{ }\gamma_{ } ,{{ \Gamma }}_{42} = 0.025{ }\gamma_{ } ,{{ \Gamma }}_{43} = 0.05\gamma_{ }$ $,{{ \Gamma }}_{31} = 0.1\gamma$,${ },{{ \Gamma }}_{41} = 0.01\gamma$ and $\gamma_{21} = 1{ }\gamma_{ } ,\gamma_{31} = 0.6{ }\gamma_{ } ,\gamma_{41} = 0.1\gamma_{ }$, respectively^47^. Note that the maximum value of the entanglement for the N-level quantum system is $DEM_{max} = \log_{2} N$. For the proposed four-level quantum dot molecule, the maximum value of the expected entanglement must be $DEM_{max} = \log_{2} 4 = 2$. We first examine the effect of quantum tunneling effect on quantum dot-photon entanglement to obtain the optimum values of $T_{A}$ and $T_{B}$. Thus, the three-dimensional entanglement diagram as a function of tunneling effects is plotted in Fig. 2a. We observe that for $T_{A} = 2{\upgamma },$ and $T_{B} = 7{\upgamma },{ }$ the maximum entanglement reaches DEM $\cong 1.97$. These tunneling effects can be controlled by the applied voltage to the quantum dot molecule^48^. Therefore, the applied voltage can directly control quantum dot-photon entanglement. Physically this is an important mechanism, where the quantum coherence is produced just by the tunneling effect that is controlled by the gate voltage.

We also propose the optimum value of ${\Omega }_{1}$ and ${\Omega }_{2}$ in Fig. 2b. It is observed that for $T_{A} = 2{\upgamma },$ and $T_{B} = 7{\upgamma }$, the DEM will be maximized for ${\Omega }_{1} = 3{ }\gamma$ and ${\Omega }_{2} = 7.5{ }\gamma .$

This is due to the fact that the maximum entanglement can be reached by evenly population distribution in four-level of the proposed quantum dot system (Fig. 2c). Then, this leads to maximum quantum dot-photon entanglement, as can be viewed in Fig. 2a,b. Note that amount of entanglement is limited by $0 \le DEM \le log_{2} N,$ where $N = 4$ represents the dimension of the Hilbert space H. Thus, for the maximally mixed state with $\rho = \frac{1}{{\text{N}}}{\text{I}}$, where ${\text{I}}$ denote an identity matrix^49^, the DEM will be maximized.

**Figure 3:**
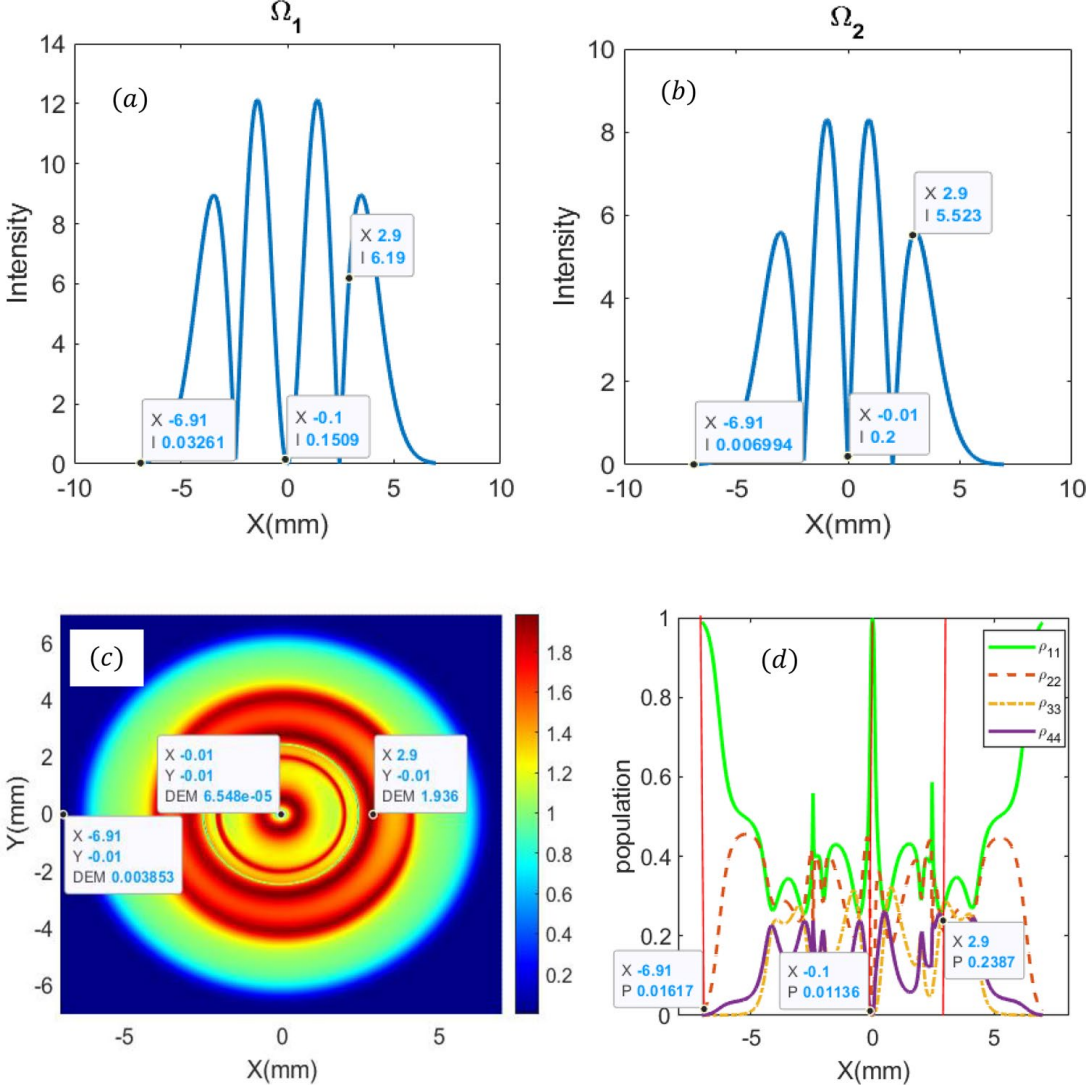
Intensity profiles of applied LG fields $\Omega_{1}$ (**a**) and $\Omega_{2}$ (**b**). DEM density plots as a function of x–y (**c**), and population distribution as a function of x (y = 0) (**d**). Parameters are $T_{A} = 2{\upgamma }$, and $T_{B} = 7{\upgamma }$, $p = 1,{\text{w}}_{0} = 2\;{\text{mm }},\;{\text{C}}_{{\text{p}}}^{{\text{l}}} = 1,{\uplambda } = 532 \times 10^{ - 9} \;{\text{m}},{\text{I}}_{0} = 10{ }\gamma$, ${\updelta } = {\Delta }_{1} = {\Delta }_{2} = 0{ }$ and ${\upomega }_{43} = 0.6{ }\gamma$. The parameter y represents the population distribution in presented levels.

Now, we are interested in studying the spatial distribution of the DEM in the x–y plane for different intensities of the applied LG fields. We consider LG modes for the applied fields and investigate the steady-state behavior of DEM at various points. The intensity profiles of LG modes for the first field ${\Omega }_{1}$(with $l = 2$) and second field ${\Omega }_{2}$ (with $l = 1$) are displayed in Fig. 3a and b.

The corresponding density plot of DEM is also displayed in Fig. 3c. From Fig. 3c, we observe that for x = 2.9 mm and y = − 0.01 mm, the maximum DEM = 1.93 is obtained. These points coincide with optimum values of ${\Omega }_{1} = 3{ }\gamma_{ }$ and ${\Omega }_{2} = 7.5{ }\gamma_{ }$ as can be viewed by Fig. 2b. For other parameters of ${\Omega }_{1}$ and ${\Omega }_{2}$, the DEM will be decreased. As an example for x = − 6.91 mm and y = − 0.01 mm, the DEM is approaching zero, and at this point, the quantum dot-photon will be disentangled. These results are justified by the population distribution of four proposed levels as depicted in Fig. 3d. For x = 2.9 mm, a quarter of the population is approximately populated in each of the four-level, i.e. *P* = 0.238.

**Figure 4:**
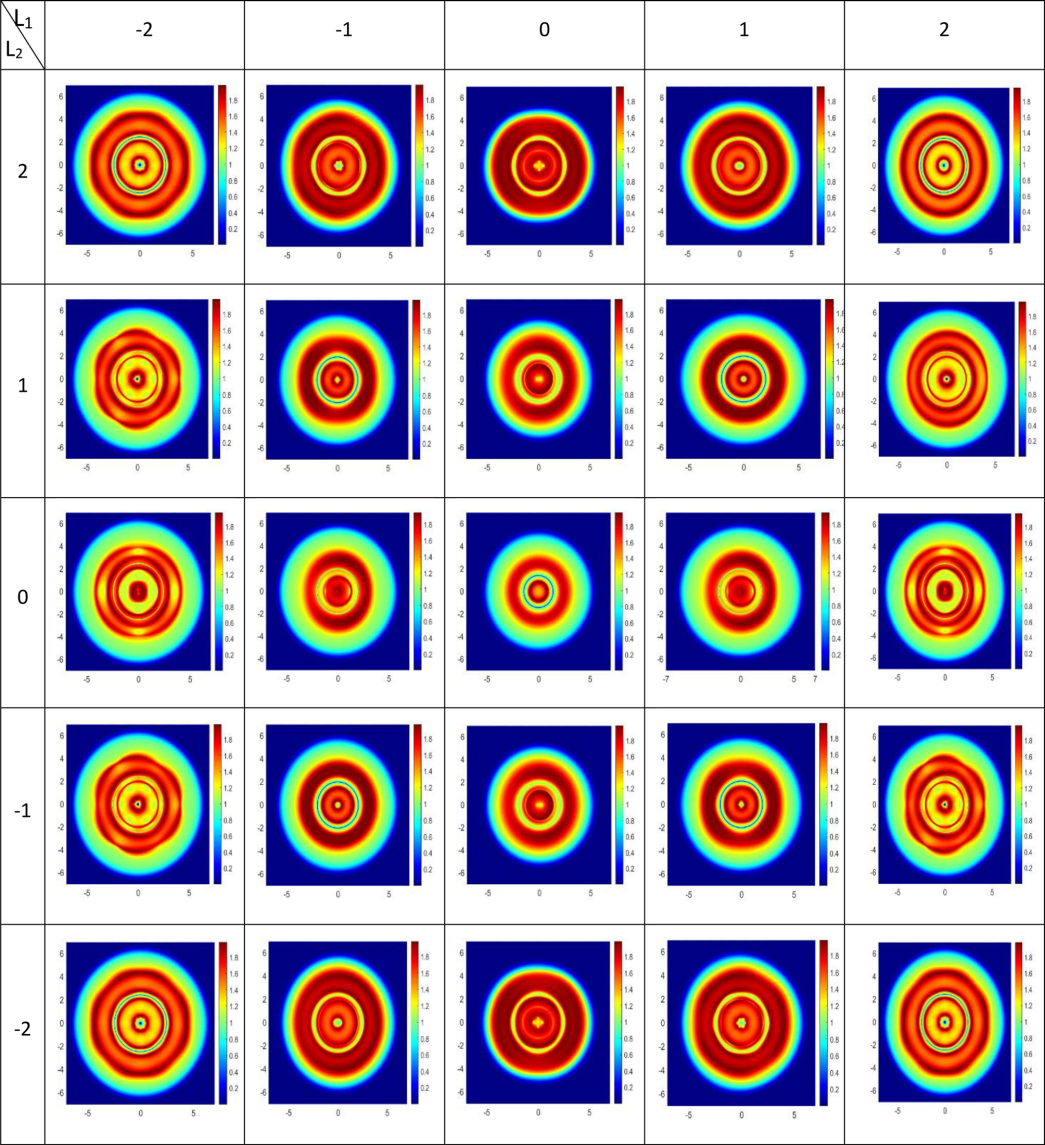
DEM density plots as a function of x and y for different modes for $l_{i}$ $= - 2, \ldots + 2$ $\left( {i = 1, 2} \right)$. Parameters are $p = 1, w_{0} = 2\;{\text{mm}},C_{p}^{l} = 1,\lambda = 532 \times 10^{ - 9} \;{\text{m}},{\text{ I}}_{0} = 10 \gamma ,\;\delta = {\Delta }_{1} = {\Delta }_{2} = 0,\;\omega_{43} = 0.6 \gamma ,\;T_{A} = 2{\upgamma }\;{\text{and}}\;T_{B} = 7{\upgamma }$.

**Figure 5:**
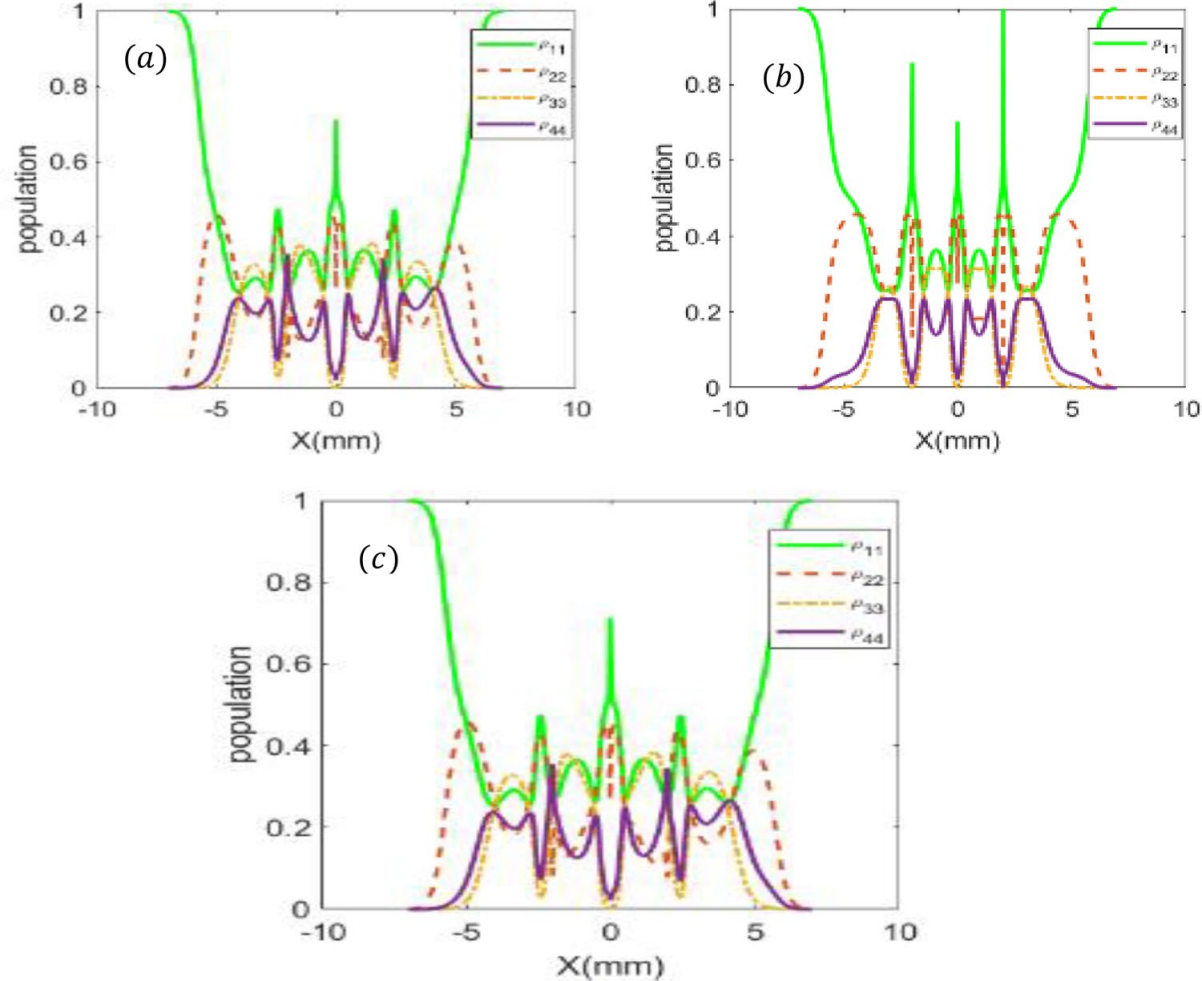
Steady-state behavior of population versus x (y = 0) for *l*_2_ = 2 and *l*_1_ =  − 1 (a), *l*_2_ = 1 and *l*_1_ = 1 (b) and *l*_2_ =  − 2 and *l*_1_ =  − 1 (c). Parameters are $p = 1,{\text{w}}_{0} = 2\;{\text{mm}},{\text{ C}}_{{\text{p}}}^{{\text{l}}} = 1,{\uplambda } = 532{*}10^{ - 9} \;{\text{m}},{ }I_{0} = 10{ }\gamma ,{\updelta } = {\Delta }_{1} = {\Delta }_{2} = 0,{\upomega }_{43} = 0.6{ }\gamma ,{\text{ T}}_{{\text{A}}} = 2\gamma ,\;{\text{and}}\;{\text{T}}_{{\text{B}}} = 7\gamma.$

Figure 4 shows the spatial distribution of DEM for various modes of LG beams, i.e. $l_{i}$ $= - 2, \ldots + 2$ $\left( {i = 1,{ }2} \right)$. For a certain mode, the maximum DEM depends on the intensity profile of the LG modes. It is different for various presented modes. The corresponding population distribution is also displayed in Fig. 5, where the population is equally distributed in four-level, DEM reaches its maximum. However, for unequal distribution of population, the quantum dot and its spontaneous emission photon is will be disentangled.

**Figure 6:**
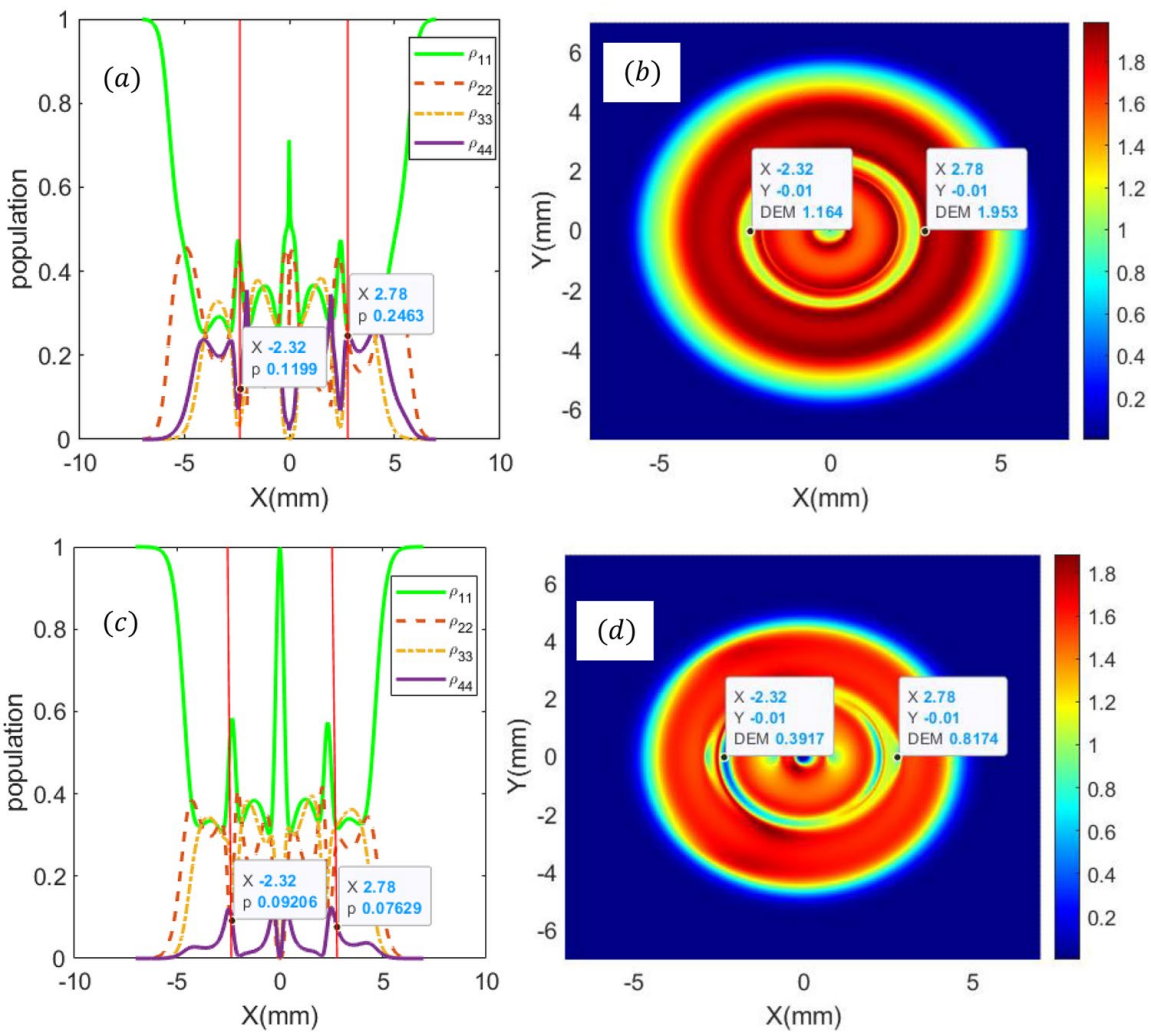
Steady-state population distribution as a function of x (y = 0) (**a**, **c**), and DEM density plots as a function of x and y (**b**, **d**) for ${\text{T}}_{{\text{A}}} = 2{\upgamma },\;{\text{and}}\;{\text{T}}_{{\text{B}}} = 7{\upgamma }$ (**a**, **b**) and ${\text{T}}_{{\text{A}}} = 6{\upgamma },\;{\text{and}}\;{\text{T}}_{{\text{B}}} = 1{\upgamma }$. Other parameters are ${\text{l}}_{2} = - 2,{\text{ l}}_{1} = - 1{ },p = 1, w_{0} = 2\;{\text{mm}},C_{p}^{l} = 1,\lambda = 532 \times 10^{ - 9} {\text{m}}, I_{0} = 10 \gamma , \delta = {\Delta }_{1} = {\Delta }_{2} = 0,\;{\text{and}}\;\omega_{43} = 0.6 \gamma$.

For further discussion of the tunneling rate between neighbor quantum dots on quantum dot-photon entanglement, we show the DEM density plot for various tunneling rates in Fig. 6. For $T_{A} = 2{\upgamma }$ and $T_{B} = 7{\upgamma }$ at x = 2.78 mm and y = − 0.01 mm, and x = − 2.32 mm and y = − 0.01 mm, the population of the four presented levels are equally distributed (Fig. 6a), and the DEM at these points are maximum (Fig. 6b). So, the quantum dot and its spontaneous emission photon are entangled. In fact, a large tunneling effect between QD2 and QD3 populates level $\left| 4 \right\rangle$ leading to the equality population distribution in four presented levels. This may also lead to strong quantum dot-photon entanglement as can be expected by $0 \le DEM \le log_{2} N,$ where $N = 4$.

However, for $T_{A} = 6{\upgamma }$ and $T_{B} = 1{\upgamma }$ and at the same points, the population are not equally distributed in four proposed levels as can be viewed in Fig. 6c. It is clear that the two strong coupling LG fields along with the tunneling rate $T_{A}$ lead to population trapping in three levels $\left| 1 \right\rangle$, $\left| 2 \right\rangle$, $\left| 3 \right\rangle$; thus the population in level 4 tends to zero. This may lead to disentanglement between the quantum dot and its spontaneous emission photon (Fig. 6d). These results show that the tunneling rates have a direct effect on population distribution and also on the spatial distribution of the quantum dot-photon entanglement.

## Conclusion

The entanglement between the triple quantum dot molecule and its spontaneous emission field is theoretically investigated by the LG fields. It is observed that the degree of entanglement and its position-dependent distribution are affected by the angular momentum of the LG lights. Thus, the position-dependent distribution of the DEM for various angular momentums of the LG fields is demonstrated. It is also shown that the spatial distribution of entanglement can be controlled by the tunneling rates between the two neighboring quantum dots. The presented results can be utilized in optical communications and information storage via preparing high-dimensional Hilbert space.

## Data Availability

There is no supplementary data for manuscript. Correspondance and requests for materials should be addressed to Y.D.
